# Effect of hyperlipidemia on the outcome of *in vitro* fertilization in non-obese patients with polycystic ovary syndrome

**DOI:** 10.3389/fendo.2023.1281794

**Published:** 2023-11-14

**Authors:** Fang Yang, Jin-Chun Lu, Tao Shen, Yi-Han Jin, Yuan-Jiao Liang

**Affiliations:** Center for Reproductive Medicine, Zhongda Hospital, Southeast University, Nanjing, Jiangsu, China

**Keywords:** polycystic ovary syndrome, hyperlipidemia, *in vitro* fertilization, pregnancy outcome, non-obese

## Abstract

**Introduction:**

It is little known whether hyperlipidemia alone has adverse effects on the outcome of *in vitro* fertilization (IVF) in patients with polycystic ovarian syndrome (PCOS).

**Methods:**

The PCOS patients with body mass index (BMI) < 30 kg/m^2^ were performed IVF or intracytoplasmic sperm injection treatment, including 208 fresh cycles and 127 frozen embryo transfer (FET) cycles. All the patients were divided into hyperlipidemia and control groups, and embryo quality and pregnancy outcomes between the two groups were compared.

**Results:**

In the fresh cycles, total gonadotropin dosage in the control group was significantly lower than that in the hyperlipidemia group, and serum estradiol levels on trigger day were reversed (*P* < 0.05). The embryo fragment score was positively correlated with serum low-density lipoprotein level (*r* = 0.06, *P* < 0.05) and negatively with serum high-density lipoprotein (HDL) and lipoprotein A levels (*r* = -0.489 and -0.085, *P* < 0.01). Logistic regression analysis found that HDL was beneficial for clinical pregnancy (OR = 0.355, 95% *CI*: 0.135-0.938, *P* < 0.05). In the FET cycles, there were no differences in pulse index, systolic/diastolic ratio and serum estradiol and progesterone levels between the two groups, but resistance index in the hyperlipidemia group was significantly higher than that in the control group (*P* < 0.05).

**Conclusion:**

Hyperlipidemia may increase the dosage of gonadotropin and have adverse effect on the embryo quality, endometrial receptivity, and clinical outcomes of lean PCOS patients. It is recommended that the non-obese patients with hyperlipidemia and PCOS perform lipid-lowering treatment before undergoing embryo transfer.

## 1 Introduction

In women of reproductive age, polycystic ovarian syndrome (PCOS) is the most prevalent endocrine condition and is characterized by hyperandrogenism, recurrent anovulation, and polycystic ovaries. A portion of PCOS is frequently linked to metabolic abnormalities such as obesity, insulin resistance (IR), poor glucose tolerance, lipid metabolic diseases, and others. Among these features obesity associated with IR is a major trigger of these disorders. Numerous researches in recent years have revealed that IR and obesity are associated with particular reproductive health issues, such as reduced clinical pregnancy rates in assisted reproductive technology (ART) cycles (1). However, there are few reports on the effects of dyslipidemia, especially in non-obese patients with PCOS, on the outcomes of assisted reproduction. It has been demonstrated that dyslipidemia in PCOS patients may exist without obesity (2), but it can occasionally exacerbate metabolic abnormalities such as obesity, IR, and others. About 70% of obese patients with PCOS have lipid abnormalities, and about 20% of lean PCOS individuals have impaired lipid metabolic disorders (3, 4). Some studies have proved that hyperlipidemia affected pregnancy outcomes of ART, such as implantation rate, clinical pregnancy rate, and miscarriage rate (5), which might be a result of alterations in the microenvironment of oocytes and endometrium brought on by lipids. Our study aimed to determine whether dyslipidemia affected the embryo quality and pregnancy outcome of non-obese PCOS patients undergoing *in vitro* fertilization (IVF) or intracytoplasmic sperm injection (ICSI), which might indirectly reflect the oocyte quality and endometrial receptivity.

The ART cycle includes fresh embryo transfer and frozen embryo transfer (FET) cycles. Considering the many differences between fresh and FET cycles, such as the higher dosage of gonadotropin (Gn), different ovarian stimulation protocols, higher estrogen levels on the human chorionic gonadotropin (HCG) trigger day, and unfrozen embryos in fresh cycles, this study investigated the fresh and FET cycles separately and compared the effects of dyslipidemia on their pregnancy outcomes. Meanwhile, considering that lipids are the substrate of steroid hormones and that abnormal lipid metabolism may have an impact on endogenous hormones, this study only selected the hormone replacement therapy (HRT) protocol for the FET cycle, which may minimize the impact of confounding factors on the results by supplementing exogenous hormones as the endometrial preparation protocol.

## 2 Materials and methods

### 2.1 Patients

The clinical data of patients undergoing conventional IVF or ICSI treatment in Center for Reproductive Medicine of Zhongda Hospital during October 2017 and June 2021 were analyzed retrospectively. The implementation of IVF or ICSI was approved by the Reproductive Medicine Ethics Committee of Zhongda Hospital affiliated to Southeast University (Reproduction No. 2015-1), and all patients signed the informed consent. The inclusion criteria of the patients included: (1) females aged < 35 years old, (2) body mass index (BMI) < 30 kg/m^2^, and (3) patients diagnosed with PCOS. The diagnostic criteria for PCOS were based on the unified standards formulated by the Rotterdam International Conference in 2003 (6). The patients with any two of the following three conditions were diagnosed with PCOS: (1) infrequent ovulation or anovulation, (2) hyperandrogenism or clinical manifestations of high blood androgen, and (3) ultrasound findings of polycystic ovaries in one or two ovaries, 12 or more follicles with a diameter of 2-9 mm, and/or ovarian volume ≥ 10 ml after exclusion of other etiologies such as congenital adrenal hyperplasia, androgen-secreting tumors, Cushing syndrome, 21-hydroxylase-deficient nonclassic adrenal hyperplasia, androgenic/anabolic drug use or abuse. Exclusion criteria included: (1) women with endocrine or metabolic diseases such as thyroid dysfunction, hyperprolactinemia, type 2 diabetes mellitus, and cardiovascular disease, (2) oocyte donation cycles, and (3) chromosome abnormality or other genetic mutations. All the patients undergoing IVF or ICSI treatments were divided into dyslipidemia (hyperlipidemia) and normal (control) groups. The diagnostic criteria of dyslipidemia were based on the 2016 guidelines for the prevention and treatment of dyslipidemia in Chinese adults (7). Dyslipidemia was defined as having at least one of the following indexes: total cholesterol (TC) ≥ 5.18 mmol/L or ≥ 200 mg/dL, low-density lipoprotein cholesterol (LDL-C) ≥ 3.37 mmol/L or ≥ 130 mg/dL, high-density lipoprotein cholesterol (HDL-C) < 1.04 mmol/L or < 40 mg/dL, and triglyceride (TG) ≥ 1.7 mmol/L or ≥ 150.62 mg/dL. The patients underwent fresh embryo transfer or frozen embryo transfer (FET) for the first time were recruited for the study. A total of 208 fresh cycles and 127 FET cycles were included. The study conforms to the WMA Declaration of Helsinki.

### 2.2 Stimulation protocols for fresh cycles

Gonadotropin-releasing hormone (GnRH) antagonist and long GnRH agonist (GnRH-a) protocols were applied for controlled ovarian stimulation (COS). The initial gonadotropin dose was determined according to the patient’s age, body weight, number of antral follicles, etc. Stimulation was monitored using estradiol concentrations, together with ultrasound measurements of follicle numbers and diameters. When at least 2 follicles were 18 mm or 3 follicles were 17 mm in diameter, 0.2 mg GnRH-a or 5000-10000 U human chorionic gonadotropin (HCG) were injected for triggering. Oocyte retrieval was performed 36 hours after HCG was injected through the transvaginal route with an ultrasound guidance. Embryo transplantation was performed 3 days after ovum pick-up (OPU) on the basis of exact situations such as estradiol concentration and endometrial thickness. One to two embryos with best quality were transferred into the uterus, and then the patients were given routine corpus luteum support. The serum HCG levels of the patients were detected 14 days after embryo transfer, and serum HCG level > 50 U/L was regarded as biochemical pregnancy. B ultrasound examination was done 4 weeks after embryo transfer, and clinical pregnancy was considered if the gestational sac was found.

### 2.3 Endometrial preparation protocols for the FET cycle

All patients in the FET cycle used HRT for endometrial preparation. First, 4 mg of oral estradiol valerate (Progynova; Bayer, Germany) was administered per day from day 2–4 of the menstrual cycle. The patients were evaluated by transvaginal ultrasound 7 days later to adjust the dosage of estradiol based on the endometrial thickness. A supplementation of vaginal estradiol was added if the endometrial thickness continued to be unsatisfactory. When the endometrial thickness reached 8 mm, and serum progesterone level was below 1.5 ng/mL, intramuscular administration of 40 mg progesterone (Zhejiang Xianju Pharmaceutical Co., Ltd, Taizhou, Zhejiang, China) or vaginal supplementation with 90 mg progesterone (8% Crinone, Merck-Serono, Germany) was added. The parameters of resistance index (RI), pulse index (PI) and systolic/diastolic ratio (S/D) were measured on the day of progesterone administration using Voluson S8 color doppler ultrasonic diagnostic apparatus (GE Ultrasound Korea, Ltd.). One to two embryos with good quality were transferred into the uterus, and then the patients were given routine corpus luteum support. The serum HCG levels of the patients were detected 14 days after embryo transfer. If the serum HCG level was higher than 50 U/L, the patient was regarded as a biochemical pregnancy. Clinical pregnancy was confirmed by ultrasound, and one or two gestational sacs were visible approximately 4 weeks after embryo transfer.

### 2.4 Assessment of embryo quality

Embryo quality was evaluated on day 3 by assessing the embryo cell number and embryo fragment score (EFS). EFS was graded on the basis of the percentage of fragmentation as follows: < 5% of fragmentation, score 4; 5%–10% of fragmentation, score 3; 11%–25% of fragmentation, score 2; 26%–50% of fragmentation, score 1; and ≥ 51% of fragmentation, score 0 (8). Good quality embryos were defined as having over 6 cells with relatively equal sized blastomeres and less than 25% of fragmentation (EFS 2-4).

### 2.5 Laboratory analysis

Commercially available kits for the determinations of serum follicle-stimulating hormone (FSH), luteinizing hormone (LH), testosterone (T), estradiol (E2), progesterone, anti-Müllerian hormone (AMH) and insulin were purchased from Abbott Laboratories, Inc. USA, and serum FSH, LH, T, E2, progesterone, AMH and insulin levels were determined by chemiluminescence assay using an automated Abbott Architect i1000 system (Abbott Laboratories, Inc., USA). Commercially available kits for the determinations of TG, TC, LDL-C, HDL-C, and lipoprotein A were purchased from Shanghai Zhicheng Biotechnology Co., Ltd., China. Calibration and quality control products were purchased from Randox Laboratories Ltd., Northern Ireland, United Kingdom. The determinations of serum lipids were carried out using a Beckman Coulter AU5800 automatic biochemistry analyzer (Beckman Coulter, Inc., USA).

### 2.6 Statistical analysis

Statistical analysis was performed with SPSS 22.0 (SPSS Inc., Chicago, IL, USA). The measurement data were first performed by one-sample nonparametric tests (Kolmogorov-Smirnov test) to determine whether they were normal distribution. The data conforming to the normal distribution were expressed as mean ± standard deviation (x ± s), and those conforming to the non-normal distribution were expressed as median [P_25_, P_75_]. The comparisons between the control and hyperlipidemia groups were analyzed by independent samples *t*-test for normal distribution data or Mann-Whitney *U* test for non-normal distribution data. The count data were presented as percentages, and the comparisons between the control and hyperlipidemia groups were analyzed by the χ2 test. The standard errors (*SE*) for percentages in two samples were calculated using the following formulas (9): the observed percentage (*p*) in the combined samples = (*n*1*p*1 + *n*2*p*2)/(*n*1 + *n*2), and *SE* = [*p*(100-p)(1/*n*1 + 1/*n*2)]^1/2^. Among them, *n*1 and *n*2 were the sample size of two groups, respectively, and *p*1 and *p*2 were the percentage of two groups, respectively. The Spearman rank correlation coefficients between EFS and lipids levels were calculated to evaluate the correlations of lipids levels with embryo quality. The multiple logistic regression analysis was used to analyze the impacts of blood lipids on the clinical outcomes of non-obese PCOS patients. They all performed the 2-sided test. *P* ≤ 0.05 was considered to be statistically significant.

## 3 Results

### 3.1 Comparisons of basic clinical information between the control and hyperlipidemia groups in the non-obese PCOS patients undergoing fresh cycle transplantation

The basic clinical information of the PCOS patients undergoing fresh cycle transplantation was presented in **Table 1**. There were no statistical differences in the age, body mass index (BMI), number of antral follicles (AFC), fasting blood-glucose (FBG), insulin, FSH, LH, testosterone, and AMH (*P* > 0.05) except serum lipid levels such as TG, TC, HDL, LDL, and lipoprotein A (*P* < 0.05) between hyperlipidemia and control groups.

**Table 1:** Comparisons of basic clinical information between the control and hyperlipidemia groups in the non-obese PCOS patients undergoing fresh cycle transplantation.

Indexes	Control (*n* = 113)	Hyperlipidemia (*n* = 95)	*P-value*
Age (year)	28.7 ± 3.1	29.4 ± 3.1	0.08
BMI (kg/m^2^)	23.0 ± 2.1	23.4 ± 2.1	0.22
Basal FSH (mIU/ml)	6.2 ± 1.4	6.0 ± 1.6	0.42
Basal LH (mIU/ml)	7.9 ± 3.7	8.2 ± 3.7	0.55
T (ng/ml)	0.66 ± 0.38	0.59 ± 0.59	0.42
AMH (ng/ml)	10.7 ± 4.1	9.6 ± 4.7	0.07
AFC (*n*)	25.4 ± 6.9	25.5 ± 6.0	0.89
FBG (mmol/L)	5.2 ± 0.5	5.3 ± 0.6	0.13
Insulin (pmol/L)	97.9 ± 40	118.4 ± 65.9	0.18
TG (mmol/L)	1.1 [0.83, 1.39]	2.43 [1.47, 3.44]	<0.01
TC (mmol/L)	4.4 ± 1.0	5.2 ± 1.3	<0.01
HDL (mmol/L)	1.54 [1.26, 2.65]	1.18 [1.00, 1.53]	<0.01
LDL (mmol/L)	2.6 ± 0.6	3.0 ± 0.8	<0.01
Lipoprotein A (mmol/L)	61.0 [40.0, 127.0]	112.0 [57.0, 255.0]	<0.01

PCOS, polycystic ovarian syndrome; BMI, body mass index; FSH, follicle stimulating hormone; LH, luteinizing hormone; T, testosterone; AMH, anti-Müllerian hormone; AFC, antral follicle count; FBG, fasting blood glucose; TC, total cholesterol; TG, triglyceride; HDL, high-density lipoprotein cholesterol; LDL, low-density lipoprotein cholesterol. The measurement data were first performed by one-sample nonparametric tests (Kolmogorov-Smirnov test) to determine whether they were normal distribution. The data conforming to the normal distribution were expressed as mean ± standard deviation (x ± s), and those conforming to the non-normal distribution were expressed as median [P_25_, P_75_]. The comparisons between the control and hyperlipidemia groups were analyzed by independent samples t-test for normal distribution data or Mann-Whitney U test for non-normal distribution data. A P-value ≤ 0.05 was considered statistically significant.

### 3.2 Comparisons of COS protocols and clinical outcomes of assisted pregnancy between the control and hyperlipidemia groups in the non-obese PCOS patients undergoing fresh cycle transplantation

The COS protocols and clinical outcomes of the non-obese PCOS patients undergoing fresh cycle transplantation were presented in **Table 2**. There were no significant differences in the Gn usage time, endometrial thickness, progesterone level on trigger day, oocytes retrieved, normal fertilization rate, good quality embryos rate, implantation rate, clinical pregnancy rate, and miscarriage rate between hyperlipidemia and control groups. However, the total Gn dosage in the hyperlipidemia group was significantly higher than that in the control group, while the estradiol level on trigger day in the hyperlipidemia group was significantly lower than that in the control group.

**Table 2:** Comparisons of COS protocols and clinical outcomes of assisted pregnancy between the control and hyperlipidemia groups in the non-obese PCOS patients undergoing fresh cycle transplantation.

Indexes	Control (*n* = 113)	Hyperlipidemia (*n* = 95)	*P-value*
Gn usage time (days)	8.8 ± 1.2	9.1 ± 1.4	0.13
Gn dosage (IU)	1740.9 ± 449	2125.6 ± 407	<0.01
Initial dose of Gn (IU)	207.5 ± 49.6	213.2 ± 56.9	0.43
COS protocols (*n*, %)			0.53
GnRH agonist	37 (32.7)	35 (36.8)	
GnRH antagonist	76 (67.3)	60 (63.2)	
Endometrial thickness (mm)	11.1 ± 2.2	11.5 ± 2.8	0.25
E2 on trigger day (pg/ml)	5365.0 [4113.0, 8179.5]	4532.0 [3470.0, 6584.5]	<0.01
P on trigger day (ng/ml)	1.2 ± 1.0	1.3 ± 0.8	0.32
Oocytes retrieved (*n*)	14.1 ± 3.9	13.4 ± 4.5	0.25
Normal fertilization rate	0.63 ± 0.17	0.66 ± 0.19	0.28
Good quality embryos rate	0.58 ± 0.19	0.59 ± 0.18	0.97
Clinical pregnancy rate (*n*, %)	17/36 (47.6)	13/28 (46.4)	0.95
Implantation rate (*n*, %)	21/44 (47.7)	15/40 (37.5)	0.34
Miscarriage rate (*n*, %)	2/17 (11.8)	2/13 (15.4)	0.77

COS, controlled ovarian stimulation; PCOS, polycystic ovarian syndrome; Gn, gonadotropin; GnRH, gonadotropin-releasing hormone; E2, estradiol; P, progesterone. Clinical pregnancy rate refers to the number of cycles achieving clinical pregnancy/the number of embryo transfer cycles; Implantation rate refers to the number of gestational sacs/the number of transplanted embryos; Miscarriage rate refers to the number of miscarriage cycles/the number of clinical pregnancy cycles. The measurement data were first performed by one-sample nonparametric tests (Kolmogorov-Smirnov test) to determine whether they were normal distribution. The data conforming to the normal distribution were expressed as mean ± standard deviation (x ± s), and those conforming to the non-normal distribution were expressed as median [P_25_, P_75_]. The comparisons between the control and hyperlipidemia groups were analyzed by independent samples t-test for normal distribution data or Mann-Whitney U test for non-normal distribution data. The count data were presented as percentages, and the comparisons between the control and hyperlipidemia groups were analyzed by the χ2 test. The standard errors (SEs) of the clinical pregnancy rate, implantation rate and miscarriage rate between the two groups were 1.73, 1.43, and 1.34, respectively. A P-value ≤ 0.05 was considered statistically significant.

### 3.3 Correlations of lipid levels with embryo quality in the non-obese PCOS patients undergoing fresh cycle transplantation

To further investigate the effects of lipid levels on embryo quality, we analyzed the correlations of lipid levels with EFS, and found that there were no significant correlations between EFS and TG or TC, but there were a positive correlation between EFS and LDL (*r* = 0.06, *P* = 0.015) and negative correlations between EFS and HDL or lipoprotein A (*r* = -0.489, *P* < 0.01; *r* = -0.085, *P* < 0.01) (**Table 3**).

**Table 3:** Correlations of lipid levels with EFS in the non-obese PCOS patients undergoing fresh cycle transplantation (*n* = 208).

Variable	EFS (*r*)	*P-value*
TG	−0.013	0.597
TC	0.042	0.088
HDL	−0.489	<0.01
LDL	0.06	0.015
Lipoprotein A	−0.085	<0.01

PCOS, polycystic ovarian syndrome; EFS, embryo fragment score; TC, total cholesterol; TG, triglyceride; HDL, high-density lipoprotein cholesterol; LDL, low-density lipoprotein cholesterol. The correlations of lipid levels with EFS were analyzed by the Spearman rank correlation. A P-value ≤ 0.05 was considered statistically significant.

### 3.4 Multiple logistic regression analysis between blood lipids and the clinical outcomes of non-obese PCOS patients undergoing fresh cycle transplantation

The multiple logistic regression analysis between blood lipids and the clinical outcomes of non-obese PCOS patients undergoing fresh cycle transplantation was shown in **Table 4**. The results showed no any correlation between blood lipids and clinical outcomes, as shown by the crude odd ratios (ORs). However, after correcting the Gn dosage and estradiol on trigger day, HDL was shown to be a protective factor for clinical pregnancy (adjusted OR [AOR] = 0.355, 95% *CI*: 0.135-0.938, *P* = 0.037) and TC might be a potential risk factor (AOR = 4.072, 95% *CI*: 0.989-16.761, *P* = 0.052). It is indicated that HDL is beneficial for clinical pregnancy and TC may be harmful to clinical pregnancy.

**Table 4:** Multiple logistic regression analysis between blood lipids and the clinical outcomes of non-obese PCOS patients undergoing fresh cycle transplantation.

Variables	Pregnancy rate		Implantation rate		Miscarriage rate	
	Crude OR (95% *CI*)	Adjusted OR (95% *CI*)	Crude OR (95% *CI*)	Adjusted OR (95% *CI*)	Crude OR (95% *CI*)	Adjusted OR (95% *CI*)
TG	0.823 (0.529-1.280) *P* = 0.387	0.742 (0.430-1.282) *P* = 0.284	0.944 (0.653-1.364) *P* = 0.757	0.807 (0.518-1.259) *P* = 0.345	1.046 (0.418-2.617) *P* = 0.923	1.383 (0.420-4.466) *P* = 0.588
TC	2.537 (0.700-8.246) *P* = 0.122	4.072 (0.989-16.761) *P* = 0.052	1.435 (0.733-2.811) *P* = 0.292	2.544 (0.873-7.410) *P* = 0.087	0.988 (0.212-4.597) *P* = 0.988	0.973 (0.117-8.069) *P* = 0.980
HDL	0.540 (0.246-1.185) *P* = 0.125	0.355 (0.135-0.938) *P* = 0.037	0.646 (0.341-1.222) *P* = 0.179	0.541 (0.257-1.138) *P* = 0.105	1.207 (0.173-8.443) *P* = 0.850	1.380 (0.158-12.072) *P* = 0.771
LDL	0.821 (0.180-3.737) *P* = 0.799	0.575 (0.102-3.244) *P* = 0.531	1.186 (0.404-3.483) *P* = 0.756	0.731 (0.175-3.065) *P* = 0.669	0.497 (0.038-6.456) *P* = 0.593	0.402 (0.022-7.369) *P* = 0.539
Lipoprotein A	0.999 (0.997-1.002) *P* = 0.693	1.000 (0.997-1.002) *P* = 0.730	1.000 (0.997-1.002) *P* = 0.790	1.000 (0.997-1.002) *P* = 0.826	1.006 (0.991-1.020) *P* = 0.427	1.006 (0.991-1.021) *P* = 0.432

TG, triglyceride; TC, total cholesterol; HDL, high-density lipoprotein cholesterol; LDL, low-density lipoprotein cholesterol. The multiple logistic regression analysis was conducted using pregnancy rate, implantation rate, or miscarriage rate as dependent variables and TG, TC, HDL, LDL, and lipoprotein A as covariates. The results were expressed in odd ratio (OR) and 95% confidence interval (CI). Crude OR and adjusted OR referred to uncorrected and corrected Gn dosage and estradiol on trigger day, respectively. A P-value ≤ 0.05 was considered statistically significant.

### 3.5 Comparisons of basic clinical information and pregnancy outcomes between the control and hyperlipidemia groups in the non-obese PCOS patients undergoing FET

The basic clinical information and pregnancy outcomes of non-obese PCOS patients undergoing FET were shown in **Table 5**. Serum lipid levels, including TC, TG, LDL, and HDL, in the hyperlipidemia group were significantly higher than those in the control group (*P* < 0.01). However, there were no significant differences in the age, BMI, FBG, serum insulin level, serum P and E2 levels on the day of progesterone administration, PI, S/D, endometrial thickness, transferred embryos, implantation rate, clinical pregnancy rate, and miscarriage rate (all *P* > 0.05) except RI (*P* = 0.002) between the hyperlipidemia and control groups.

**Table 5:** Comparisons of basic clinical information and pregnancy outcomes between control and hyperlipidemia groups in the PCOS patients undergoing FET.

Indexes	Control (*n* = 76)	Hyperlipidemia (*n* = 51)	*P-value*
Age (years old)	29.2 ± 2.9	29.8 ± 2.1	0.23
BMI (kg/m^2^)	23.6 ± 3.0	24.3 ± 1.8	0.12
FBG (mmol/L)	5.06 ± 0.45	5.05 ± 0.6	0.98
Insulin (pmol/L)	87.4 ± 36.4	94.2 ± 33.4	0.29
TG (mmol/L)	1.22 [0.91, 1.56]	2.36 [1.74, 2.80]	<0.01
TC (mmol/L)	4.47 [4.07, 5.44]	5.04 [4.73, 5.81]	<0.01
HDL (mmol/L)	1.55 [1.36, 1.69]	1.40 [1.01, 1.49]	<0.01
LDL (mmol/L)	2.58 ± 0.64	3.13 ± 0.68	<0.01
Lipoprotein A (mmol/L)	86.5 [51.5, 152.2]	112.0 [82.0, 143.0]	0.04
Endometrial thickness (mm)	10.86 ± 1.61	11.36 ± 1.56	0.087
RI	0.82 ± 0.05	0.84 ± 0.05	0.002
PI	2.30 ± 0.47	2.35 ± 0.41	0.49
S/D	6.21 ± 1.62	6.42 ± 1.65	0.46
Serum E2 on the day of progesterone administration (pg/ml)	273.2 ± 93.9	276.4 ± 104.2	0.86
Serum P on the day of progesterone administration (ng/ml)	0.58 ± 0.24	0.63 ± 0.24	0.19
Transferred embryos (*n*)	1.6 ± 0.49	1.5 ± 0.50	0.54
Clinical pregnancy rate (*n*, %)	44/76 (57.9)	27/51 (52.9)	0.58
Implantation rate (*n*, %)	57/119 (47.9)	31/77 (40.3)	0.29
Miscarriage rate (*n*, %)	5/44 (11.4)	3/27 (11.1)	0.97

PCOS, polycystic ovarian syndrome; FET, frozen embryo transfer; BMI, body mass index; FBG, fasting blood glucose; TC, total cholesterol; TG, triglyceride; HDL, high-density lipoprotein cholesterol; LDL, low-density lipoprotein cholesterol; RI, resistance index; PI, pulse index; S/D, systolic/diastolic ratio; E2, estradiol; P, progesterone. Clinical pregnancy rate refers to the number of cycles achieving clinical pregnancy/the number of embryo transfer cycles; Implantation rate refers to the number of gestational sacs/the number of transplanted embryos; Miscarriage rate refers to the number of miscarriage cycles/the number of clinical pregnancy cycles. The measurement data were first performed by one-sample nonparametric tests (Kolmogorov-Smirnov test) to determine whether they were normal distribution. The data conforming to the normal distribution were expressed as mean ± standard deviation (x ± s), and those conforming to the non-normal distribution were expressed as median [P_25_, P_75_]. The comparisons between the control and hyperlipidemia groups were analyzed by independent samples t-test for normal distribution data or Mann-Whitney U test for non-normal distribution data. The count data were presented as percentages, and the comparisons between the control and hyperlipidemia groups were analyzed by the χ2 test. The standard errors (SEs) of the clinical pregnancy rate, implantation rate and miscarriage rate between the two groups were 1.35, 0.97, and 0.82, respectively. A P-value ≤ 0.05 was considered statistically significant.

### 3.6 Multiple logistic regression analysis between blood lipids and the clinical outcomes of non-obese PCOS patients undergoing FET

The multiple logistic regression analysis between blood lipids and the clinical outcomes of non-obese PCOS patients undergoing FET was shown in **Table 6**. The results showed no any correlation between blood lipids and clinical outcomes, whether corrected or uncorrected for RI.

**Table 6:** Multiple logistic regression analysis between blood lipids and the clinical outcomes of non-obese PCOS patients undergoing FET.

Variables	Pregnancy rate		Implantation rate		Miscarriage rate	
	Crude OR (95% *CI*)	Adjusted OR (95% *CI*)	Crude OR (95% *CI*)	Adjusted OR (95% *CI*)	Crude OR (95% *CI*)	Adjusted OR (95% *CI*)
TG	1.048 (0.707-1.554) *P* = 0.815	1.058 (0.712-1.572) *P* = 0.700	0.903 (0.651-1.252) *P* = 0.541	0.899 (0.646-1.249) *P* = 0.524	0.709 (0.340-1.476) *P* = 0.358	0.725 (0.344-1.529) *P* = 0.399
TC	1.054 (0.561-1.979) *P* = 0.870	1.043 (0.554-1.961) *P* = 0.897	1.163 (0.710-1.906) *P* = 0.549	1.166 (0.711-1.913) *P* = 0.542	0.978 (0.252-3.796) *P* = 0.974	0.975 (0.241-3.935) *P* = 0.972
HDL	1.185 (0.533-2.635) *P* = 0.677	1.141 (0.506-2.570) *P* = 0.750	0.960 (0.527-1.749) *P* = 0.895	0.973 (0.530-1.786) *P* = 0.930	1.134 (0.083-15.411) *P* = 0.925	0.964 (0.061-15.188) *P* = 0.979
LDL	1.188 (0.518-2.724) *P* = 0.685	1.250 (0.533-2.932) *P* = 0.608	0.962 (0.502-1.843) *P* = 0.908	0.945 (0.486-1.837) *P* = 0.866	0.598 (0.100-3.568) *P* = 0.573	0.671 (0.105-4.311) *P* = 0.675
Lipoprotein A	0.997 (0.992-1.002) *P* = 0.210	0.997 (0.992-1.002) *P* = 0.202	0.997 (0.993-1.000) *P* = 0.073	0.997 (0.993-1.000) *P* = 0.074	1.005 (0.993-1.018) *P* = 0.434	1.005 (0.992-1.018) *P* = 0.427

TG, triglyceride; TC, total cholesterol; HDL, high-density lipoprotein cholesterol; LDL, low-density lipoprotein cholesterol. The multiple logistic regression analysis was conducted using pregnancy rate, implantation rate, or miscarriage rate as dependent variables and TG, TC, HDL, LDL, and lipoprotein A as covariates. The results were expressed in odd ratio (OR) and 95% confidence interval (CI). Crude OR and adjusted OR referred to uncorrected and corrected resistance index (RI), respectively. A P-value ≤ 0.05 was considered statistically significant.

## 4 Discussion

PCOS is the most common endocrine disorder in reproductive-aged women. Women with PCOS have increased risk of metabolic disorders such as IR, hyperandrogenism, obesity and hyperlipidemia. Researchers have confirmed the effects of obesity and IR on fertility. A compensatory hyperinsulinemic state caused by obesity and PCOS may disrupt endometrial homeostasis and result in insulin receptors decrease and defective decidualization (10). Hyperandrogenism could affect the window of implantation by decreasing the expression levels of *HOXA10* and *WT1* genes and influencing endometrial decidualization. Cui et al. (11) verified that the obese PCOS patients undergoing IVF/ICSI treatment had lower clinical pregnancy rate. Dyslipidemia often coexists with obesity and IR, and some studies have found that the incidence of hyperlipidemia in PCOS patients increased by 16.1% when compared with the general population (12). However, hyperlipidemia may exist without obesity in PCOS patients, and sometimes it can aggravate obesity, IR and a variety of metabolic abnormalities, which have serious effects on cardiovascular system and fertility (2). Therefore, we speculate that hyperlipidemia may affect the pregnancy outcome of ART. However, the relationship between serum lipids and the pregnancy outcomes of IVF/ICSI in PCOS patients is rarely reported.

The present study was to elucidate the characteristics of serum lipids and their effects on oocyte and endometrium in non-obese women with PCOS undergoing IVF/ICSI. The women chosen for the retrospective analysis had BMI below 30 kg/m^2^, and their hyperandrogenism were pretreated.

Our investigation found that the non-obese PCOS patients with hyperlipidemia required higher Gn dosage during ovulation induction, which was consistent with previous research results (13). However, the specific reasons are still unclear. Some researchers found that the serum level of sex hormone binding globulin in the patients with hyperlipidemia decreased, and that free testosterone levels increased, which would reduce the sensitivity of ovary to Gn (14). It was also found that tumor necrosis factor (TNF) was involved in the regulation of oocyte maturation and apoptosis, and that high TNFα levels might lead to the arrest of oocyte maturation and chromosome abnormality, and reduce the sensitivity of ovary to Gn (15). While, the increase of blood lipids especially triglyceride was positively correlated with TNFα (16).

An analysis of 1394 treatment cycles of 943 patients found that daily dose of Gn and total Gn dosage were negatively correlated with the number of oocytes, implantation rate, clinical pregnancy rate, and live-birth rate, which recommended that daily Gn dose is preferably less than 450 IU or total Gn dosage less than 3000 IU/cycle (17). Another study found that daily Gn dose exceeding 300 IU was significantly associated with a lower live birth rate (18). Excessive Gn exposure may lead to an unfavorable endometrium or a detrimental metabolic environment (19). It is speculated that increasing Gn dosage may cause high circulating progesterone level and a premature luteinization of the endometrium (20). It is also well established that the use of exogenous Gn affects the endometrium and can result in functional genomic disorder of the endometrium (21). Therefore, high Gn dosage in non-obese PCOS patients with hyperlipidemia may also affect endometrial receptivity.

In addition, numerous studies have demonstrated that the metabolic problems related to hyperlipidemia, such as hyperinsulinemia and aberrant adipokines, may affect embryonic development and endometrial receptivity (22). Some studies have demonstrated that hyperlipidemia has an adverse effect on embryo quality, and that the exact mechanisms need to be further verified. Some researches showed that hyperlipidemia could produce a large number of reactive oxygen species (ROS), and then destroy the morphology and integrity of endoplasmic reticulum of granulosa cells. All these changes would disturb the maturation of oocytes and reduce the blastocyst formation rate (8, 23, 24). Some animal experiments found that high fat diets might cause important defects and chromosome dislocations during meiosis of oocytes (25).

In our study, although there were no significant differences in the normal fertilization rate and good quality embryos rate between the control and hyperlipidemia groups, the embryo fragmentation score (EFS) was positively correlated with LDL and negatively with HDL and lipoprotein A (*P* < 0.05). Moreover, the multiple logistic regression analysis showed that HDL was beneficial for clinical pregnancy (AOR = 0.355, 95% *CI*: 0.135-0.938, *P* = 0.037) and TC may be a potential risk factor for clinical pregnancy (AOR = 4.072, 95% *CI*: 0.989-16.761, *P* = 0.052). Some studies also demonstrated that the levels of follicular fluid and plasma HDL were negatively correlated with embryo fragmentation (26). It was reported that transferring embryos with more fragments had lower implantation and pregnancy rates than transferring embryos with minimal fragments (27). Browne et al. (28) confirmed that HDL exhibited important cytoprotective effect on oocytes and granulosa cells around them. The most compelling evidence for the importance of HDL in mammalian oocyte development and competence was exemplified by studies of SR-BI KO mice (29). The SR-BI known as HDL receptor could facilitate the uptake of cholesterol for steroidogenesis. Oocyte and embryo morphology were disrupted in SR-BI KO female mice. However, it is unclear how HDL works to safeguard embryonic growth. Some researchers thought that it might be associated with antioxidation. The HDL particles include phospholipids, triglycerides and proteins such as PON1, which play an antioxidant role and would protect embryos from the damage of ROS (30). Theoretically, follicular fluid HDL is a filtration byproduct of human plasma. The plasma HDL level might partly reflect the HDL level in follicular fluid. However, the HDL particles in follicles contain less cholesterol and rich phospholipids relative to those in serum. Moreover, the HDL particles in follicles are heterogeneous in structure, and it is unclear which type of HDL is primarily related to embryo fragmentation. Kim et al. (31) found that higher concentrations of follicular fluid HDL subfractions in the large and medium-sized particles were associated with poorer embryo quality. Therefore, we speculate that HDL plays a protective role in oocyte and embryo development, but which kind of HDL is necessary still needs to be further explored.

Although we found a harmful effect of hyperlipidemia on embryos in fresh cycles, there were no differences in clinical pregnancy rate and miscarriage rate between hyperlipidemia and control groups. This may be attributed to the fact that the best embryos with few fragments in the first cycle were transplanted. Considering that serum E2 levels on trigger day in the control group were significantly higher than that in the hyperlipidemia group, and that serum E2 level was crucial for embryo implantation, we further investigated the FET outcomes of the two groups. All the patients undergone the FET for the first time received HRT. There were no significant differences in serum E2 levels and endometrial thickness on the day of progesterone administration. Meanwhile, all the transferred embryos were of good quality, similar to the fresh transfer cycle. The results showed that there were no significant differences in the implantation rate, pregnancy rate, and miscarriage rate between hyperlipidemia and control groups, but the resistance index (RI) was significantly different.

Uterine blood supply mainly comes from the uterine artery and its branches, including the arcuate artery supplying the myometrium and the spiral artery supplying the endometrium. During the embryo implantation stage, the blood supply to the uterus alters to ensure adequate blood supplementation. Kim et al. (32) used Doppler ultrasonography to measure endometrial and subendometrial vascularity on the day of HCG administration during IVF-ET, and found that endometrial and subendometrial vascularity in the pregnancy group was significantly higher than that in the control group. It was reported that a successful FET cycle was related to increased blood flow to the endometrium, which boosted endometrial receptivity (33). However, the results of this study showed that the difference of RI was not enough to lead to the difference of implantation rate. Subendometrial vascularity indexes such as vascularity index (VI) and vascularity flow index (VFI) are more accurate than uterus artery indexes including RI, PI and S/D. Some studies found that there were no differences in uterus artery indexes such as PI, RI and S/D scores between pregnancy and non-pregnancy groups (32, 34). In recent studies subendometrial vascularity indexes were more prone to be selected to detect endometrial receptivity. However, due to the significant technical requirements for measuring subendometrial blood vessels, we only measured the uterus artery indexes. This is one of the limitations of our study. This study is only conducted in one institute, which requires more data to confirm. Another limitation of this study is the limited sample size. Analyzing the FET cycle and fresh cycle separately may further reduce the testing power of the used samples. In the future, we will increase the sample size to further confirm the impact of hyperlipidemia on the clinical outcomes of ART in PCOS patients and explore its possible mechanisms.

## 5 Conclusions

In conclusion, our study found that the total Gn dosage in the hyperlipidemia group was significantly higher than that in the control group, and that the embryo fragment score was positively correlated with LDL and negatively with HDL and lipoprotein A. Moreover, HDL was beneficial for clinical pregnancy. In addition, in FET cycles, the resistance index in the hyperlipidemia group was significantly higher than that in the control group. It is indicated that hyperlipidemia may increase the dosage of gonadotropin and have adverse effect on the embryo quality, endometrial receptivity, and clinical outcomes of lean PCOS patients. It is recommended that the non-obese patients with hyperlipidemia and PCOS perform lipid-lowering treatment before undergoing embryo transfer.

## Data availability statement

The raw data supporting the conclusions of this article will be made available by the authors, without undue reservation.

## Ethics statement

The studies involving humans were approved by The clinical data of patients undergoing conventional IVF or ICSI treatment in our hospital during October 2017 and June 2021 were analyzed retrospectively. The implenmentation of IVF or ICSI was approved by the Reproductive Medicine Ethics Committee of Zhongda Hospital affiliated to Southeast University (Reproduction No. 2015-1), and all patients signed the informed consent. The studies were conducted in accordance with the local legislation and institutional requirements. The human samples used in this study were acquired from primarily isolated as part of your previous study for which ethical approval was obtained. Written informed consent for participation was not required from the participants or the participants’ legal guardians/next of kin in accordance with the national legislation and institutional requirements.

## Author contributions

FY: Data curation, Formal Analysis, Writing – original draft. J-CL: Conceptualization, Data curation, Writing – review & editing. TS: Data curation, Writing – original draft. Y-HJ: Data curation, Writing – original draft. Y-JL: Conceptualization, Resources, Writing – review & editing.

## Glossary

**Table:**

PCOS	polycystic ovarian syndrome
BMI	body mass index
IVF	*in vitro* fertilization
ICSI	intracytoplasmic sperm injection
FET	frozen embryo transfer
IR	insulin resistance
ART	assisted reproductive technology
TC	total cholesterol
LDL-C	low-density lipoprotein cholesterol
HDL-C	high-density lipoprotein cholesterol
TG	triglyceride
RI	resistance index
PI	pulse index
S/D	systolic/diastolic ratio
GnRH	Gonadotropin-releasing hormone
Gn	gonadotropin
GnRH-a	GnRH agonist
COS	controlled ovarian stimulation
HCG	human chorionic gonadotropin
OPU	ovum pick-up
EFS	embryo fragment score
FSH	follicle-stimulating hormone
LH	luteinizing hormone
T	testosterone
E2	estradiol
P	progesterone
AMH	anti-Müllerian hormone
AFC	antral follicles
FBG	fasting blood-glucose
TNF	tumor necrosis factor
